# Comprehensive analysis of the chloroplast genome structure and phylogeny of *Glochidion puberum* (L.) Hutch.

**DOI:** 10.1080/23802359.2026.2680739

**Published:** 2026-06-01

**Authors:** Kaiming Gao, Yang Ni, Jiahua Chen, Xinmin Pan, Guoan Shen

**Affiliations:** a Center for Bioinformatics, Institute of Medicinal Plant Development, Chinese Academy of Medical Sciences & Peking Union Medical College, Beijing, China; b School of Chemistry and Chemical Engineering, Guangdong Pharmaceutical University, Zhongshan, Guangdong, China; c Guangxi Hongyao Biotechnology Co., Ltd, Laibin, Guangxi, China

**Keywords:** *Glochidion puberum*, chloroplast genome, phylogenetic analysis, phyllanthaceae

## Abstract

*Glochidion puberum* (L.) Hutch., a member of the family Phyllanthaceae, is widely distributed in China and possesses both medicinal and ornamental value. Despite its ecological and economic significance, the complete chloroplast genome of *G. puberum* had not been reported, and its phylogenetic placement within the genus *Glochidion* has remained unresolved. In this study, we assembled and annotated the complete chloroplast genome of *G. puberum* for the first time. The chloroplast genome is 157,133 bp in length, exhibiting an overall GC content of 37% and containing 129 genes. The result of phylogenetic analysis revealed a close evolutionary relationship among *G. puberum*, *G. hirsutum*, and *G. chodoense* based on complete chloroplast genome. These findings provide comprehensive genomic data for *G. puberum* and offer new perspectives on the phylogenetic relationships and evolutionary position in the broader Phyllanthaceae family.

## Introduction

*Glochidion puberum* (L.) Hutch., 1916 belongs to the family Phyllanthaceae, which is classified under the genus *Glochidion* and is widely distributed in China and also recorded in Japan (Bouman et al. 2022). *Glochidion puberum* is a traditionally used medicinal plant with significant pharmacological properties. The chemical components of the genus *Glochidion* mainly include triterpenoid saponins, sesquiterpenoids, glycosides, and alkaloids (Sandhya et al. 2010). The pharmacological effects of the genus *Glochidion* cover many aspects such as anti-inflammatory (Hossen et al. 2021), anti-tumor (Tian et al. 2023), and anti-bacterial (Sandhya et al. 2010). In traditional Chinese medicine, *G. puberum* is utilized for the treatment of dysentery, jaundice, leukorrhea, common cold, sore throat, toothache, carbuncles, furuncles, and rheumatic arthralgia (Hu et al. 2004). In the previous study of *G. puberum*, most of them mainly focus on chemical composition, and pharmacological effects (Sandhya et al. 2010, Tian et al. 2023). However, there has been no reported chloroplast genome for *G. puberum*.

In this study, the chloroplast genome sequence of *G. puberum* was obtained using the data from Illumina HiSeq 2500 platform. Subsequently, we analyzed the sequence characteristics, gene composition, and phylogenetic relationships. This study aims to analyze the characteristics of the chloroplast genome of *G. puberum* and elucidate its evolutionary status within the family Phyllanthaceae, thereby providing new insights for phylogenetic research on this group.

## Materials and methods

**Figure 1.**
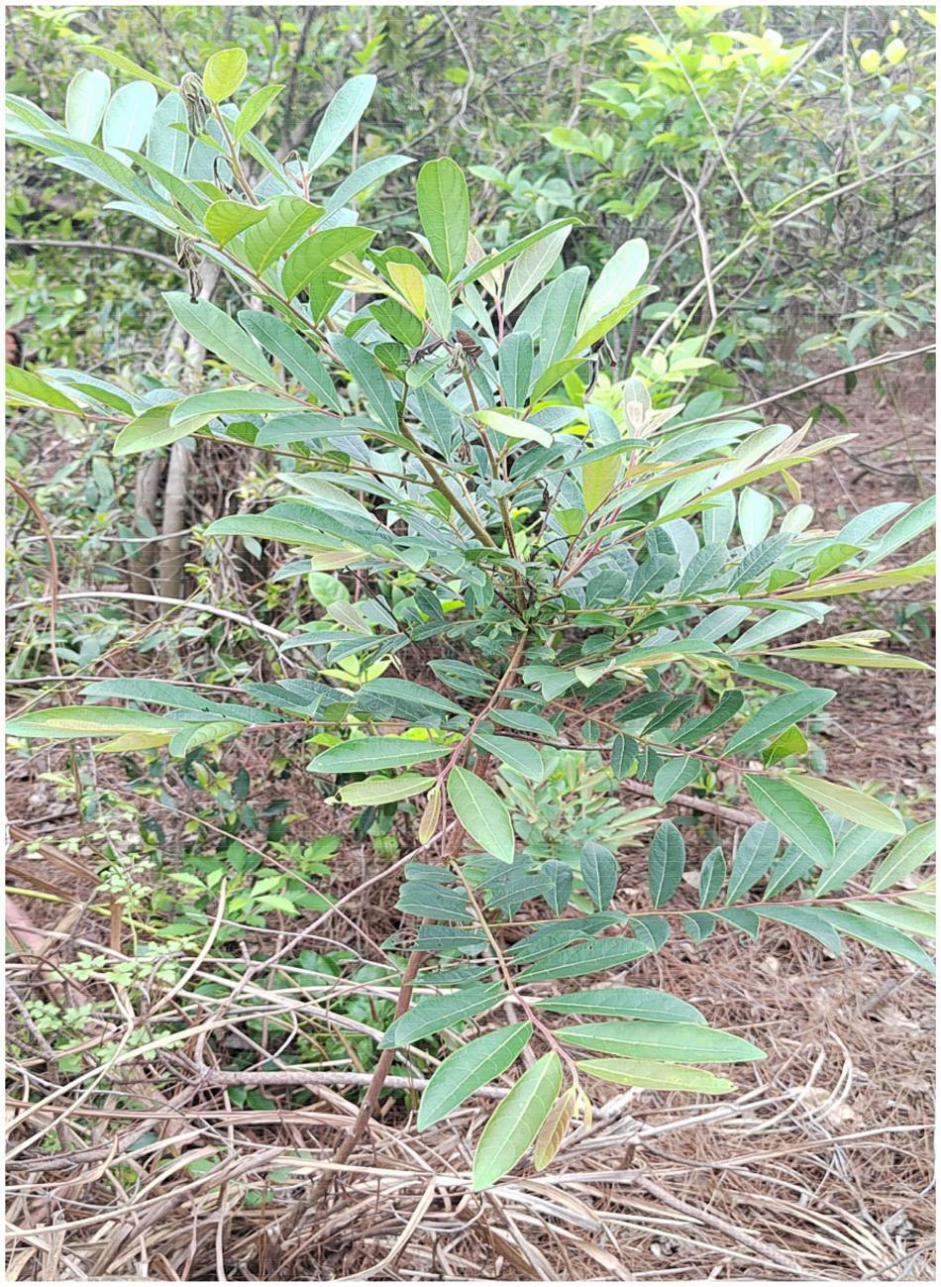
Photograph of *G. puberum.* (the photo was taken by Zhaosheng Pang and authorized for use. It is the first publication of this study.). *G. puberum* is an upright, multi-branched shrub. Its leaves are alternate, mostly long-rectangular to long-elliptical, about several centimeters in length, with 5–7 pairs of distinct pinnate lateral veins and clear reticulate veins.

Fresh leaves of *G. puberum* were collected from Jinxiu Yao Autonomous County, which is situated in Laibin City, Guangxi, China (E 110°11′, N 24°8′, 500 m) and preserved in desiccant. Plants (Figure 1) were identified by Mr. Zhaosheng Pang. The sample is stored in Room 301, Shengzhi Building, Institute of Medicinal Plant Development, Chinese Academy of Medical Sciences & Peking Union Medical College (Guoan Shen: gashen@implad.ac.cn), with the certificate number JXHC050.

Total DNA was extracted using the Plant Genomic DNA Kit (Tiangen Biotech, Co., Ltd., Beijing, China) and subsequently stored in a refrigerator set at −80 °C. A DNA library containing inserts of 300 base pairs (bp) was successfully constructed. Subsequently, paired-end sequencing with a read length of 150 bp was carried out using the Illumina HiSeq 2500 platform. Quality control of raw sequencing reads was conducted using fastp (Chen et al. 2018). This process generated high-quality filtered reads by performing adapter trimming (auto-detected), removing low-quality bases (Phred score < 20), and discarding reads shorter than 50 bp. The reads were subsequently assembled through the application of the GetOrganelle toolkit (Jin et al. 2020). The annotation and visualization of the chloroplast genome were completed *via* CPGAVAS2 (Shi et al. 2019) and CPGView (Liu et al. 2023), respectively.

We conducted a phylogenetic analysis utilizing 23 chloroplast genomes from various species within the family Phyllanthaceae, and selected the *Acalypha australis* (NC_073100.1) and *Acalypha hispida* (NC_070339.1) as the outgroups. All chloroplast genome sequences were obtained from the NCBI GenBank database using their accession numbers (Table S1). Firstly, the PhyloSuite software (Zhang et al. 2020) was utilized to extract the common Coding Sequences (CDS) among these species, and 60 common protein-coding genes were selected and aligned using the MAFFT software (Katoh and Standley 2013). Subsequently, the aligned sequences were employed to build the Maximum-likelihood tree with IQ-TREE2 (Lanfear et al. 2020). The best-fit substitution model (Q.bird + F + I + G4) was selected by ModelFinder (Kalyaanamoorthy et al. 2017), and statistical support for branches was assessed with 5000 ultrafast bootstrap replicates (Hoang et al. 2018). Lastly, the phylogenetic tree was visualized using the iTOL website (Letunic and Bork 2021).

## Results

**Figure 2.**
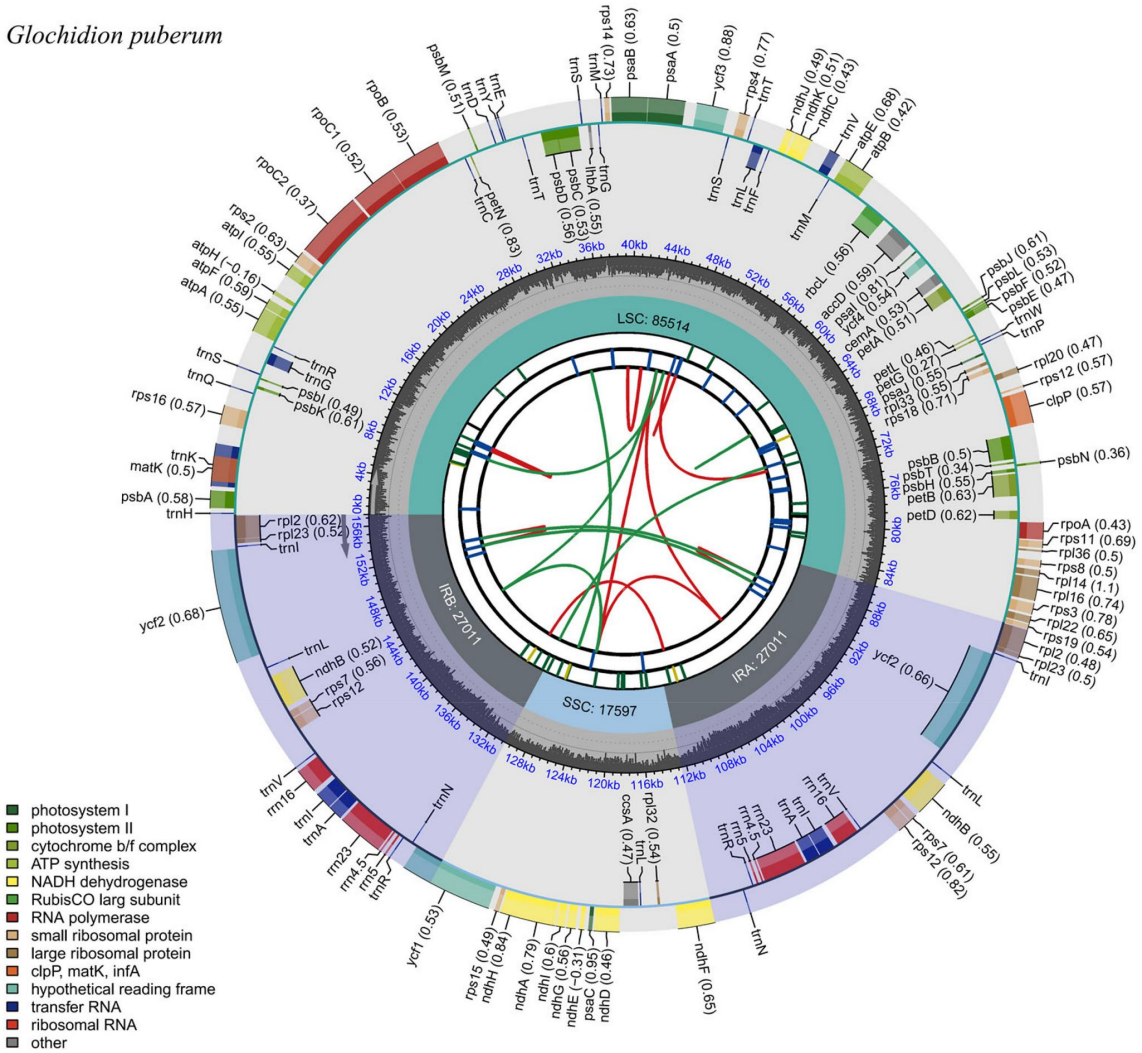
Circular map of *G. puberum* chloroplast genome, created through the CPGView platform. From the outside to the center, the first circle shows transcription directions of gene. The transcription of the inner genes occurs in a clockwise direction, while that of the outer genes proceeds in an anticlockwise direction. The potential codon usage bias is indicated in the parentheses following each gene name. The functional classification of genes is displayed in different colors in the lower left corner. The second circle represents the GC content of the chloroplast genome. The third circle shows four components of the chloroplast genome: LSC (depicted in green), SSC (shown in blue), IRA, and IRB (represented in gray). The fourth circle shows the long repeats whose location are marked by blue bars. The fifth circle depicts microsatellite sequences through the use of short bars in a variety of colors, with each color representing distinct types of repeats.

A total of 13.5 G bases data of *G. puberum* was obtained. The chloroplast genome exhibited a length of 157,133 bp. The minimal coverage depth of the chloroplast genome reached 1008× and the maximal coverage depth reached 7296× (Figure S1). The chloroplast genome exhibited a typical quadripartite structure, comprising a large single-copy region (85,514 bp), a small single-copy region (17,597 bp), and two inverted repeat regions (27,011 bp each) (Figure 2). The average guanine-cytosine content was 37%. A total of 129 functional genes have been annotated, including 84 protein-coding genes (PCGs), 37 transfer RNA genes (tRNAs), and 8 ribosomal RNA genes (rRNAs). Cis-splicing analysis (Figure S2) identified multiple genes containing introns, including *rps16*, *rpoC1*, *ycf3*, *clpP*, *petB*, *rpl16*, *rpl2*, *ndhB*, and *ndhA*. These genes exhibit typical exon-intron structures, with exons and introns distributed in varying lengths and positions across the chloroplast genome. The *rps12* gene serves as a canonical model for illustrating trans-splicing in chloroplasts (Figure S3). Its coding sequence is discontinuously distributed across distinct regions of the chloroplast genome: exon1 resides exclusively within the large single-copy (LSC) region, whereas exon2 and exon3 are located within the inverted repeat (IR) region. Due to the presence of two identical IR copies (IRa and IRb), exon2 and exon3 each exist in duplicate on the chloroplast genome.

**Figure 3.**
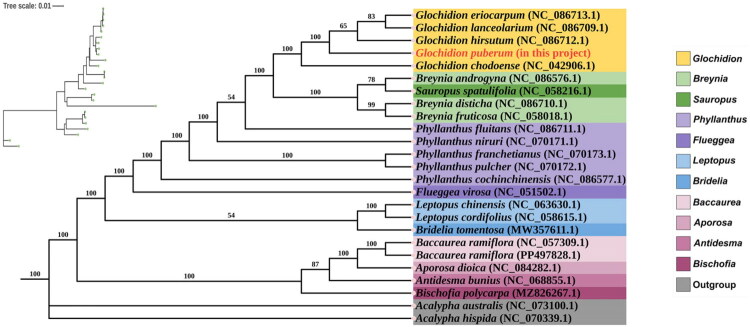
A Maximum-Likelihood Tree illustrating the relationship between *G. puberum* and 22 species from various genera in the phyllanthaceae family. *Acalypha australis* and *acalypha hispida*, belonging to the euphorbiaceae family, serve as outgroups. The values above the branches indicate the ML bootstrap support. Various genera are distinguished by different colors. Notes (Table S1): PV700501.1 (In this Project), NC_042906.1 (Cheon et al., 2019), NC_058216.1 (Cai et al., 2020), NC_086710.1 (Wei and Li 2025), NC_058018.1 (Zhou et al., 2020), NC_070173.1 (Fang et al., 2023), NC_051502.1 (Wang et al., 2020), NC_063630.1 (Zhao and Yu 2022), NC_058615.1 (Rehman et al., 2021), MW357611.1 (Chen et al., 2021), NC_073100.1 (Li et al., 2024), NC_070339.1(Dong et al., 2023). NC_086713.1, NC_086709.1, NC_086712.1, NC_086576.1, NC_086711.1, NC_070171.1, NC_070172.1, NC_086577.1, NC_057309.1, PP497828.1, NC_084282.1, NC_068855.1, and MZ826267.1 (Unpublished Public Sequences).

The phylogenetic analysis strongly supports a monophyletic clade comprising *G. eriocarpum*, *G. lanceolarium*, *G. hirsutum*, *G. puberum*, and *G. chodoense*. This close relationship implies a recent shared ancestry and may explain observed morphological similarities among these species. In contrast, the genus *Glochidion* as a whole forms a distinct lineage that is clearly divergent from other Phyllanthaceae genera (such as the genus *Breynia*, *Sauropus*, and *Phyllanthus*), confirming its genetic distinctness (Figure 3).

## Discussion and conclusion

The chloroplast genome size of *G. puberum* is 157,133 bp, which falls within the range observed among other *Glochidion* species, whose genome sizes vary from approximately 157,085 bp to 157,460 bp. This consistency suggests a relatively stable chloroplast genome size within the genus *Glochidion*. When compared to other members of the Phyllanthaceae family, the chloroplast genome sizes show greater variability, ranging from about 149,958 bp in *Bridelia tomentosa* to over 162,220 bp in *Bischofia polycarpa*. This wider variation among family members likely reflects evolutionary divergence and adaptation to distinct ecological niches.

The classification of the genus *Phyllanthus* remains contentious. Previous studies have suggested that the genera *Breynia*, *Glochidion*, and *Synostemon* are closely related to *Phyllanthus*, which has led to the proposal of a broadly defined *Phyllanthus s.l.* that includes these taxa (Hoffmann et al. 2006, Kathriarachchi et al. 2006). In contrast, other researchers have proposed a solution to strictly divide the genus *Phyllanthus* into monophyletic genera to ensure that the classification is consistent with the recent phylogenetic results (Bouman et al. 2022).

Our phylogenetic analysis based on chloroplast genomes supports a close evolutionary relationship between *Breynia* and *Phyllanthus*, which is consistent with previous molecular findings (Nguyen et al. 2023). Despite these close affinities, our results do not provide sufficient justification for merging these genera into a single taxon. From a systematic perspective, maintaining smaller, monophyletic genera can improve phylogenetic clarity and taxonomic stability (de Gasper et al. 2017). This is supported by the utility of the highly conserved chloroplast genome, which is widely used to resolve complex phylogenetic relationships and refine taxonomic frameworks (Kim et al. 2025, Ma et al. 2025). Nevertheless, this study has certain limitations, particularly limited sampling, which may restrict the resolution of deeper evolutionary divergences within the group.

Future research will aim to develop novel molecular markers derived from the chloroplast genomes of *Glochidion* species. These markers could improve phylogenetic resolution, enhance species delimitation, and facilitate marker‑assisted selection in breeding programs. Overall, our findings provide the first complete chloroplast genome sequence of *G. puberum*, elucidate its phylogenetic position within Phyllanthaceae, and offer valuable genomic resources for future phylogenetic, population genetic, and conservation studies.

## Supplementary Material

Supplemental Material

## Data Availability

The genome sequence data of this study can be accessed in GenBank with the accession number PV700501.1. Additionally, the corresponding BioProject, BioSample, and SRA numbers are PRJNA1269032, SAMN48775440, SRR33736593, respectively.

## Permission for sample collection

*G. puberum* is not classified as a protected plant species, no permits are necessary for collecting samples.
