# Supplementary material for: Comprehensive analysis of the chloroplast genome structure and phylogeny of Glochidion puberum (L.) Hutch
Source: Mitochondrial DNA B Resour. 2026 Jun 1;11(7):802–6. doi: 10.1080/23802359.2026.2680739 (PMC13228165; doi:10.1080/23802359.2026.2680739)
Supplement: Supplemental Material [file TMDN_A_2680739_SM8511.docx]

Notes (**Table S1**): PV700501.1 (In this Project), NC_042906.1 (Cheon et al., 2019), NC_058216.1 (Cai et al., 2020), NC_086710.1 (Wei et al., 2025), NC_058018.1 (Zhou et al., 2020), NC_070173.1 (Fang et al., 2023), NC_051502.1 (Wang et al., 2020), NC_063630.1 (Zhao et al., 2022), NC_058615.1 (Rehman et al., 2021), MW357611.1 (Chen et al., 2021), NC_073100.1 (Li et al., 2024), NC_070339.1(Dong et al., 2023). NC_086713.1, NC_086709.1, NC_086712.1, NC_086576.1, NC_086711.1, NC_070171.1, NC_070172.1, NC_086577.1, NC_057309.1, PP497828.1, NC_084282.1, NC_068855.1, and MZ826267.1 (Unpublished Public Sequences).

# Supplemental materials

**Table S1**. The family, genus, species, and GenBank accession numbers list of 25 species.

| **Number** | **Family** | **Genus** | **Species** | **Accession number** | **References** |
| --- | --- | --- | --- | --- | --- |
| 1 | Phyllanthaceae | *Glochidion* | *Glochidion eriocarpum* | NC_086713.1 | NA |
| 2 | Phyllanthaceae | *Glochidion* | *Glochidion lanceolarium* | NC_086709.1 | NA |
| 3 | Phyllanthaceae | *Glochidion* | *Glochidion hirsutum* | NC_086712.1 | NA |
| **4** | **Phyllanthaceae** | ***Glochidion*** | ***Glochidion puberum*** | **PV700501.1** | **In this Project** |
| 5 | Phyllanthaceae | *Glochidion* | *Glochidion chodoense* | NC_042906.1 | (Cheon et al., 2019) |
| 6 | Phyllanthaceae | *Breynia* | *Breynia androgyna* | NC_086576.1 | NA |
| 7 | Phyllanthaceae | *Sauropus* | *Sauropus spatulifolius* | NC_058216.1 | (Cai et al., 2020) |
| 8 | Phyllanthaceae | *Breynia* | *Breynia disticha* | NC_086710.1 | (Wei et al., 2025) |
| 9 | Phyllanthaceae | *Breynia* | *Breynia fruticosa* | NC_058018.1 | (Zhou et al., 2020) |
| 10 | Phyllanthaceae | *Phyllanthus* | *Phyllanthus fluitans* | NC_086711.1 | NA |
| 11 | Phyllanthaceae | *Phyllanthus* | *Phyllanthus niruri* | NC_070171.1 | NA |
| 12 | Phyllanthaceae | *Phyllanthus* | *Phyllanthus franchetianus* | NC_070173.1 | (Fang et al., 2023) |
| 13 | Phyllanthaceae | *Phyllanthus* | *Phyllanthus pulcher* | NC_070172.1 | NA |
| 14 | Phyllanthaceae | *Phyllanthus* | *Phyllanthus cochinchinensis* | NC_086577.1 | NA |
| 15 | Phyllanthaceae | *Flueggea* | *Flueggea virosa* | NC_051502.1 | (Wang et al., 2020) |
| 16 | Phyllanthaceae | *Leptopus* | *Leptopus chinensis* | NC_063630.1 | (Zhao et al., 2022) |
| 17 | Phyllanthaceae | *Leptopus* | *Leptopus cordifolius* | NC_058615.1 | (Rehman et al., 2021) |
| 18 | Phyllanthaceae | *Bridelia* | *Bridelia tomentosa* | MW357611.1 | (Chen et al., 2021) |
| 19 | Phyllanthaceae | *Baccaurea* | *Baccaurea ramiflora* | NC_057309.1 | NA |
| 20 | Phyllanthaceae | *Baccaurea* | *Baccaurea ramiflora* | PP497828.1 | NA |
| 21 | Phyllanthaceae | *Aporosa* | *Aporosa dioica* | NC_084282.1 | NA |
| 22 | Phyllanthaceae | *Antidesma* | *Antidesma bunius* | NC_068855.1 | NA |
| 23 | Phyllanthaceae | *Bischofia* | *Bischofia polycarpa* | MZ826267.1 | NA |
| 24 | Euphorbiaceae | *Acalypha* | *Acalypha australis* | NC_073100.1 | (Li et al., 2024) |
| 25 | Euphorbiaceae | *Acalypha* | *Acalypha hispida* | NC_070339.1 | (Dong et al., 2023) |

Note: NA in the Reference column indicates that the sequence was obtained through Direct Submission to GenBank and is not associated with a formal scientific publication.

**
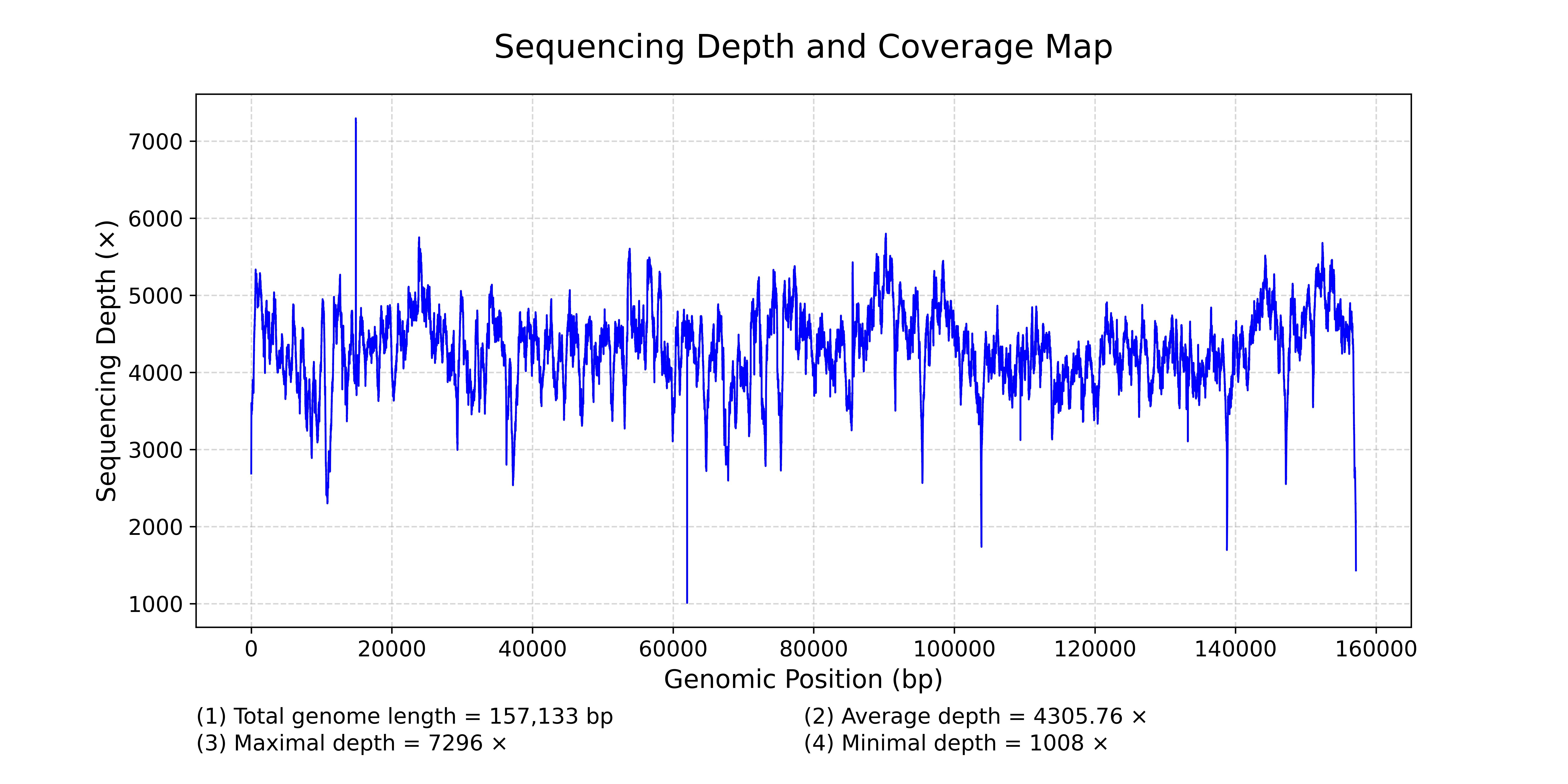
Figure S1.** The reads coverage plot of *G. puberum* chloroplast genome. The x-axis represents the position within the chloroplast genome, while the y-axis illustrates the sequencing depth.

**
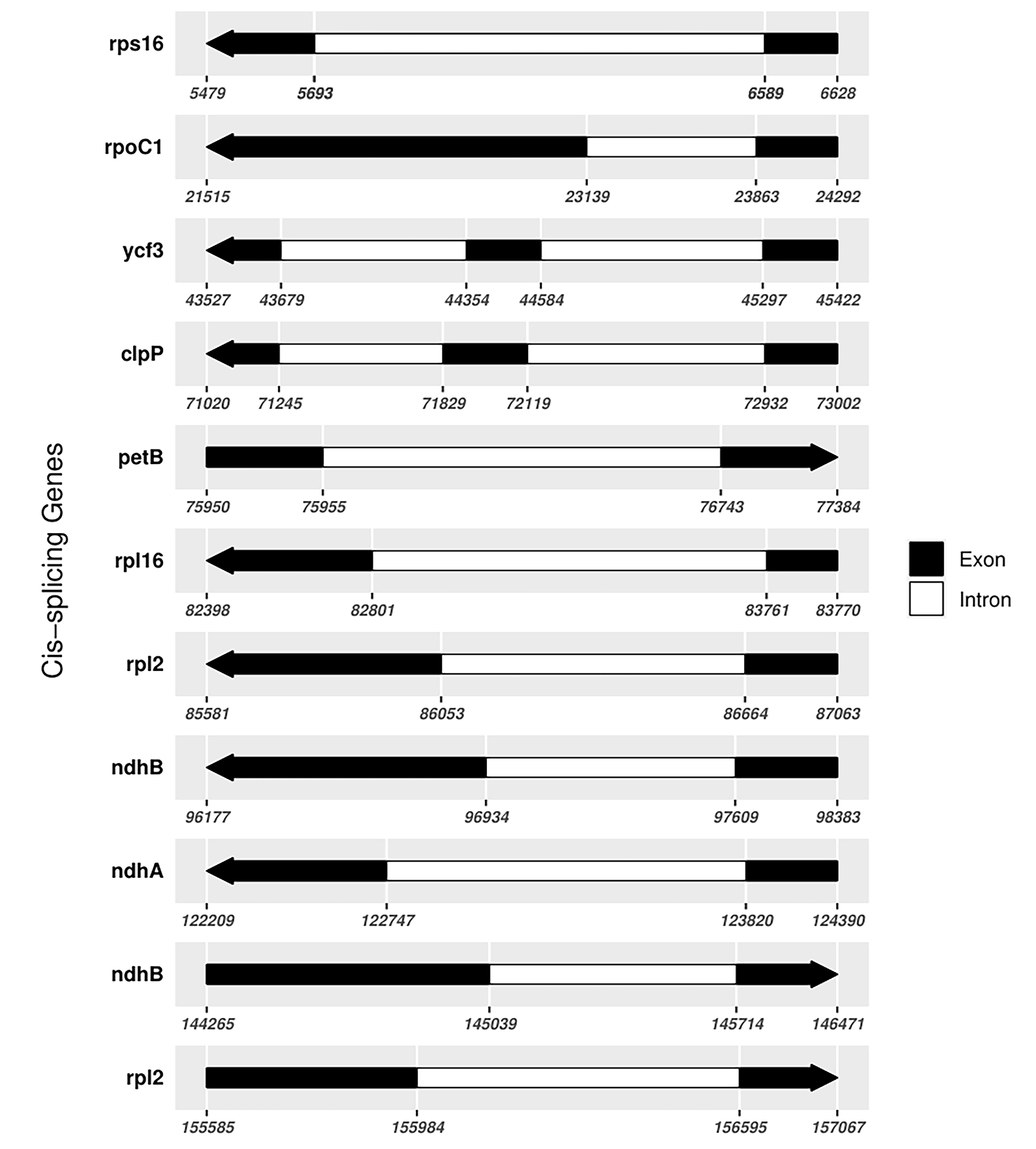
**

**Figure S2.** The diagram illustrating the cis-splicing genes within the chloroplast genome of *G. puberum*. The direction of each gene is described by the associated arrow. The gene consists of exons (black parts) and introns (white parts). The name of genes is positioned on the left. The numbers below each arrow indicate its genomic coordinates in the genome.


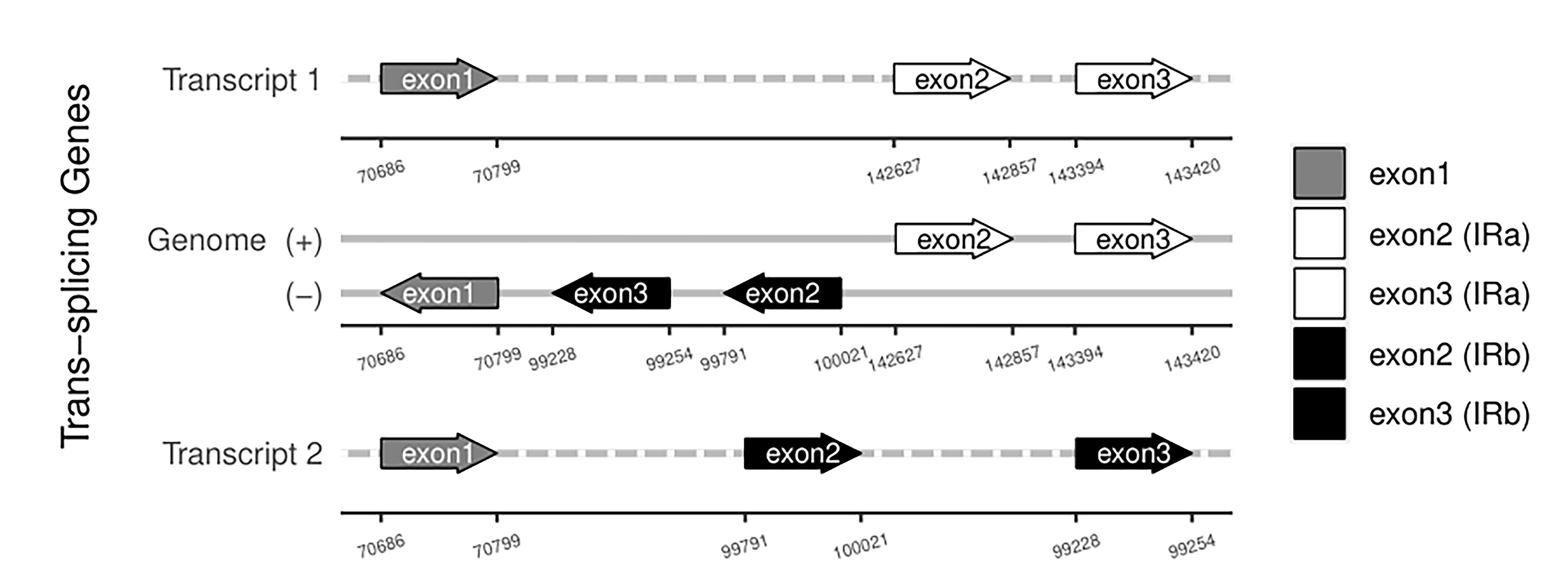


**Figure S3.** Diagram illustrating the trans-splicing genes (*rps12*) within the chloroplast genome of *G. puberum*. The gene consists of exons (arrows) and introns (lines).
